# Behavioral and Neurophysiological Aspects of Inhibition—The Effects of Acute Cardiovascular Exercise

**DOI:** 10.3390/jcm10020282

**Published:** 2021-01-14

**Authors:** Oron Levin, Yael Netz, Gal Ziv

**Affiliations:** 1 Movement Control and Neuroplasticity Research Group, Department of Kinesiology, KU Leuven, 3001 Heverlee, Belgium; oron.levin@kuleuven.be; 2 Department of Health Promotion and Rehabilitation, Lithuanian Sports University, LT-44221 Kaunas, Lithuania; 3 The Academic College at Wingate, Netanya 4290200, Israel; galziv@yahoo.com

**Keywords:** motor inhibition, cognitive inhibition, physical activity, exercise, GABA, glutamate, prefrontal-basal-ganglia pathways

## Abstract

This review summarizes behavioral and neurophysiological aspects of inhibitory control affected by a single bout of cardiovascular exercise. The review also examines the effect of a single bout of cardiovascular exercise on these processes in young adults with a focus on the functioning of prefrontal pathways (including the left dorsolateral prefrontal cortex (DLPFC) and elements of the prefrontal-basal ganglia pathways). Finally, the review offers an overview on the potential effects of cardiovascular exercise on GABA-ergic and glutamatergic neurotransmission in the adult brain and propose mechanisms or processes that may mediate these effects. The main findings show that a single bout of cardiovascular exercise can enhance inhibitory control. In addition, acute exercise appears to facilitate activation of prefrontal brain regions that regulate excitatory and inhibitory pathways (specifically but not exclusively the prefrontal-basal-ganglia pathways) which appear to be impaired in older age. Based on the reviewed studies, we suggest that future work examine the beneficial effects of exercise on the inhibitory networks in the aging brain.

## 1. Introduction

Inhibition plays an important role in the control of many cognitive and motor functions [1,2]. On a conceptual level, inhibition can be viewed as a process that limits the spreading of neural activity to or from nearby regions that are not relevant to the task at hand [3], suppresses prepotent responses, and allows withdrawing, reprogramming, or termination of voluntary movements [4,5,6] or downregulates attentional processes directed toward irrelevant stimuli [7,8]. At the cortical level, inhibition is largely mediated via activation of gamma-aminobutyric acid (GABA) receptors. GABA is a principal inhibitory neurotransmitter in the brain tissue of mammalians with a rich structural diversity of receptors and a dense representation of GABA-ergic interneurons in the neocortex (e.g., [9]). For example, GABA-ergic activity in the primary motor cortex (M1) has been reported to play a prominent role in the fine tuning of corticospinal excitability during movement initiation and movement withdrawal [2] or during movement preparation [5,10]. Deficiencies or abnormalities in GABA-ergic mediated neural mediated inhibition in M1 have been documented in many cognitive and/or movement disorders, particularly in those diseases where excess or undesired movements emerge such as dystonia [11,12] (for a review, see [13]). Finally, from a system functionality point of view, inhibition is mediated primarily by a widespread prefrontal network including the right inferior frontal gyrus (rIFG), the pre-supplementary motor area (preSMA), the left-dorsolateral prefrontal cortex (DLPFC), and the basal ganglia [14,15,16,17] (for reviews, see [18,19,20]).

Inhibitory processes can be classified as reactive (i.e., cessation of a response that is already in progress) or proactive (i.e., inhibitory control mechanisms engaged prior to the initiation of a response) [16,18,21,22]. Functional magnetic resonance imaging (fMRI) studies have shown that reactive inhibition is associated primarily with the activation of a hyperdirect pathway in which the subthalamic nucleus (STN) receives direct inputs from the rIFG and preSMA [17,18,23]. In contrast, proactive inhibition is thought to rely more on activation of indirect pathways of the basal ganglia (cortico-striato-pallido-subthalamo-nigral) [16,17,18,24,25,26] or activation of direct and indirect pathways from the left prefrontal and premotor areas to the primary motor cortices (M1) [14,15,27]. Notably, activation of all aforementioned pathways involves recruitment of prefrontal subregions which are more prone to age-related structural changes than central and posterior brain areas [28,29].

Changes in the ability to regulate response inhibition over the lifespan can be attributed partly to structural changes in white matter (WM) tracts connecting the prefrontal-basal-ganglia pathways [30,31]. More recent research using proton magnetic resonance spectroscopy (1H-MRS) and/or transcranial magnetic stimulation (TMS) revealed significant associations between impaired inhibitory control and age-related changes in the neurochemical characteristics of the prefrontal-basal ganglia network, prefrontal-premotor network, and the visuomotor network [10,32,33,34]. Studies using neuroimaging methods (e.g., fMRI) suggest that older adults can often compensate for declining performance in inhibition tasks by recruiting additional ipsilateral and contralateral brain regions mainly in the left prefrontal regions [30,35]. In line with these findings, we need to understand how compensatory recruitment of prefrontal inhibitory pathways can be augmented to preserve cognitive and motor abilities in aging. Because pharmacological approaches may have unwanted side effects, there is an interest in non-pharmacological, low-risk intervention methods such as physical activity [36].

The literature on the effects of acute exercise on cognitive performance has been growing over the past years (for a review, see [37]; see also [38] for a review on high-intensity exercise). Researchers have also explored the underlying mechanisms that mediate the relationship between acute exercise and cognitive performance (e.g., motor memory and consolidation [39] and mood and general cognition [40]). However, to the best of our knowledge, there have not been any attempts to summarize the literature on the effects of acute exercise on inhibitory control. Such a review is timely because inhibitory control is a pivotal mechanism underlying cognitive (e.g., [1,35]) and motor performance (e.g., [14,41]; for a review, see [19]). In this review, we highlight possible mechanisms that may underlie the beneficial effects of acute cardiovascular exercise on inhibitory control.

## 2. Literature Search and Characteristics of the Included Studies

### 2.1. Search Strategy

We conducted an electronic search of the literature in two databases—PubMed and Google Scholar—by using the following search terms: (“physical activity” OR aerobic OR endurance OR cycling OR swimming OR running OR jogging OR walking OR “cross country”) AND (stroop OR flanker OR go/no-go OR go/nogo OR go-no-go OR “go-no go” OR “choice reaction time” OR “choice-reaction time”) NOT (Parkinson OR Parkinson’s OR Alzheimer OR Alzheimer’s OR dementia OR “cognitive impairment” OR stroke OR schizophrenia). All behavioral tasks that were included in the search (i.e., the Stroop task, the Flanker task, choice reaction time, go/no-go, and “stop” after “go”) were expected to produce a significant recruitment of cortical and subcortical pathways (e.g., [16,17,42,43]). The search was limited to peer-reviewed articles published in the English language. Studies were included only if acute exercise was performed. In addition, lists of references from relevant studies were scanned for further sources. We conducted the initial search in May 2020. An additional search was conducted in December 2020. The search yielded 22 relevant studies [44,45,46,47,48,49,50,51,52,53,54,55,56,57,58,59,60,61,62,63,64,65], which are summarized in Table 1.

### 2.2. Sample Characteristics and Interventions

Eighteen of the 22 reviewed studies included young adults or university students (average age < 30 years). In most studies, participants underwent a cardiovascular exercise on an ergometer or a treadmill at intensities ranging from moderate to vigorous [45,46,47,49,51,52,55,56,60,64,65]. Four studies applied high intensity training protocols [54,59,61,63]. One study applied various levels of training intensity [62]. One study used bouts of resistance and aerobic exercise [53]. In one study [50], children exercised with movement games, and, in another study [44], participants performed chair exercises. Finally, in one study [48], participants cycled at 40% maximal aerobic power.

### 2.3. Behavioral Outcome Measures

Inhibition was assessed with various versions of the color-word Stroop test [44,47,53,55,57,60,61,62,64,65], the go-no/go test [45,49], the Flanker task [63], or the stop-signal test [46,48,66]. Deficits in inhibition can lead to increased conflict between competing stimuli. Such conflict increases response time and response errors. While this can be investigated by the Stroop task [67], it should be noted that age-related increases in response time could be mediated by processes unrelated to inhibition per se. For example, decreased performance of higher-order brain functions such as perception and attention are expected to hinder processing time and action selection [7]. Particularly, studies using TMS suggest that brain structures associated with action selection are also involved in regulation of inhibitory projections from prefrontal and premotor regions to M1 (e.g., [14]). These inhibitory processes are hypothesized to be particularly important during movement preparation than during movement execution (e.g., [27]). Therefore, it is expected that response errors could offer a more objective assessment of inhibitory control than response time or reaction time.

The integrity of inhibitory control could be assessed more directly with the use of go/no-go stop signal task (e.g., [20]). In this task, participants are instructed to respond to “go” cues but to withdraw their response if the “go” cue is followed by a “stop” signal ([16,32,42,68,69]; see review [70]). This task was used to measure the efficiency of the reactive inhibition process by deriving the internal reaction time to the stop signal (i.e., the stop-signal reaction time (SSRT)) as previously described, for example, by Verbruggen and Logan [70]. Findings from studies using fMRI have shown that successful reactive inhibition is associated primarily with the activation of a hyperdirect pathway in which the subthalamic nucleus (STN) receives direct inputs from RIFG and preSMA [16,17,18,23,25,26,30,42,71,72]. Importantly, the inhibitory network mediating SSRT has been shown to be susceptible to age-related WM microstructural changes (e.g., [31]) or neurometabolic changes [34]. Therefore, SSRT is considered a primary index of response inhibition and a valid behavioral marker for integrity of the prefrontal-basal ganglia network.

### 2.4. Neurophysiological Outcome Measures

#### 2.4.1. TMS-Based Outcome Measures

Four studies assessed the effects of physical activity on neurophysiological measures of inhibition [51,54,58,59]. At the cortical level, inhibition is largely mediated via activation of GABA receptors. Deficiencies or abnormalities in GABA-ergic activity have been documented in many cognitive and/or movement disorders, particularly in those diseases where excess or undesired movements emerge such as dystonia [11] and epilepsy [73] (see [13] for a review).

The activity of cortical GABA-ergic inhibitory interneurons can be monitored in-vivo with transcranial magnetic stimulation (TMS) (e.g., [74,75]; see [76] for a review). The status of intracortical inhibition within the primary motor cortex can be explored by the means of paired-pulse TMS (ppTMS), in which two separate pulses are delivered to the motor cortex through the same TMS coil at interstimulus intervals (ISI) of 2–3 ms (short-interval intracortical inhibition, SICI) or ∼100 ms (long-interval intracortical inhibition, LICI) [74,75,76]. Evidence from pharmacological studies indicate that SICI is mediated by activation of short acting GABA_A_ receptors [75,77], while LICI is believed to be mediated by slow acting GABA_B_ receptors [78,79]. Other TMS protocols (e.g., ppTMS with a subthreshold CS that precedes a suprathreshold TS by 8–30 ms) reflect the combination of GABA and glutamatergic activity including intracortical facilitation (ICF) [76,80].

Finally, the excitability of GABA_B_-ergic interneurons in M1 can also be explored by applying a single TMS pulse during the voluntary contraction of a target muscle. This produces a motor evoked potential (MEP) in the target muscle contralateral to the stimulated M1 followed by a period of suppressed EMG activity, which is termed contralateral silent period (cSP). The duration of cSPs has been reported to be prolonged by administration of GABA_B_ agonist, baclofen [74], suggesting that cSP is mediated by GABA_B_-ergic interneurons, with longer cSP durations indicating increased GABA_B_ receptor mediated signaling in M1.

#### 2.4.2. Electroencephalography-Based Outcome Measures

Four studies assessed the effects of physical activity on inhibition using electroencephalography (EEG) [45,46,47,50,63]. The influence of physical exercise on inhibitory processes associated with response preparation and response generation can be assessed, indirectly, with the analyses of event-related potentials (ERPs) (e.g., [81]). Research has focused on the no-go-N140 component elicited by the go/no-go task at Fz and Cz electrode sites. In addition, the anterior P300 ERPs during no-go and stop trials have been argued to represent core features of inhibitory process in the frontal lobe [45,82,83]. P300 amplitude also increases after moderate-intensity exercise and decreases after high-intensity exercise [84,85], suggesting that high intensity exercise may be accompanied by an overall increase in arousal. Regarding the effects of physical activity and/or aging on cognitive inhibition, studies using Stroop paradigms showed that incompatible Stroop stimuli evoked centro–parietal negative EPRs in the time range between 300 and 600 ms post stimulus with a negative peak around 450 ms (referred to as N450 component) [86]. The increase of Stroop interference in healthy older individuals has been found to be associated with an attenuation the magnitude of the N450 (e.g., [87]), suggesting that decreased magnitude of the N450 component could be used as a marker of compromised cognitive inhibition in aging. Finally, studies using frequency domain analyses have revealed that increased oscillatory activity in the beta power bands (12–30 Hz) at fronto-central electrodes was associated with successful stop trial [88]. Other EEG features such as the N1 component elicited by the stop-signal [46], the frontal-midline N200 component, or the oscillatory activity in the theta (4–8 Hz) and alpha (8–12 Hz) power bands have been identified as neurophysiological markers of performance on go/no-go, Stroop, stop signal task, or the Flanker task paradigms [82].

## 3. Effects on Behavioral and Neurophysiological Aspects of Inhibition

### 3.1. Behavioral Measures

Behavioral effects of a single bout of cardiovascular exercise on inhibitory control were studied using go/no-go [45,49,52], Stroop [44,47,53,55,57,60,61,62,64,65], Flanker [63], or stop signal test paradigms [46,48]. For the most part, these studies reported: (i) an overall pre-to-post decrease in reaction time in the go trials and missed responses on the no-go trials [45,52]; (ii) reduction in Stroop interference effects [44,47,55]; or (iii) decrease in SSRT [46,48]. Significant pre-to-post shortening of Stroop-interference reaction time and overall reduction in the performance time of the Stroop task were reported in most of the studies that used this task [44,47,53,55,60,61,64,65]. However, the positive time gains were expected to be mediated by overall improvements in executive control, rather than by exercise-induced enhancements of top-down cognitive control of inhibition. In one study [62], a trend towards an improvement on Stroop interference effects was accompanied by general improvements in a Trail Making test (TMT), suggesting an overall positive effect of exercise on executive control. In another study [53], the beneficial effect of exercise on inhibition control correlated positively with significant pre-to-post improvement of mood. Similarly, significant pre-to-post decrease of reaction time and increase of correct responses in the children’s determination test [50] may point to overall improvements in memory and attention, albeit exercise-induced gains in top-down inhibitory processes should not be excluded.

Finally, the evidence for pre-to-post decrease in anti-saccade reaction time [56] could be taken as an evidence for an exercise-based increase in attention/arousal and/or improved task-specific activity within the frontoparietal networks supporting anti-saccades. Evidence from TMS studies showed that cortical circuits connecting the posterior parietal cortex (PPC) to the premotor cortex (PMC) might be involved in sensorimotor transformations and movement preparation (e.g., [89]). These interactions could be mediated at least partly by inhibitory projections from PMC to M1 and frontal eye-field (FEF). Inhibitory projections from PC to FEF may be effective for optimizing fixation on the target during movement preparation and movement planning [90] and can partly explain the observed reduction of anti-saccade reaction time. However, Samani et al. [56] revealed no pre-to-post effect of acute exercise on anti-saccade directional errors, suggesting limited or no impact of intervention on inhibitory regulation of eye-movements.

Besides improvements in motor and cognitive functioning, acute cardiovascular exercise also promotes motor skill consolidation and off-line learning gains [59]. The mechanisms associated with these processes have been reviewed elsewhere [39] and are not discussed in the present review.

### 3.2. Neurophysiological Measures

Neurophysiological measures consisted of the N140 [45], P300 [46,63], and N450 [47] ERPs, beta-power bands of EEG signal [50] and TMS-based assessments of corticomotor excitability (CME), intracortical inhibition (SICI and LICI), and intracortical facilitation (ICF) [51,54,59].

#### 3.2.1. TMS

All studies that used TMS to examine the effects of acute exercise on cortico-cortical interactions reported a significant pre-to-post exercise effect in at least one of the tested inhibitory outcome measures [51,54,58,59]. However, findings were inconsistent. In one study [58,59] a significant reduction in SICI was found immediately after the end of exercise, suggesting post-exercise downregulation (decrease) of GABA_A_-receptor mediated inhibition. Measures of GABA_B_-receptor mediated inhibition (LICI) and non-synaptic inhibition (SICI_1 ms_) were not affected. These findings are somewhat contradictory to the findings of Singh et al. [58] who reported post-exercise reduction of GABA_A_ (SICI)- and GABA_B_ (LICI)-receptor mediated inhibition or Mooney et al. [51] who reported post-exercise reduction of LICI but not SICI. Finally, O’Leary et al. [54] reported a significant post-exercise downregulation GABA_B_- receptor mediated inhibition, expressed by reduction of LICI and decrease of contralateral silent period (cSP) duration. The latter observation was inconsistent with that of Mooney et al. [51] who reported no post-exercise changes of cSP. Factors that could account for the observed inconsistencies were exercise intensity and exercise duration. In two studies, the intervention consisted of exhaustive cycling session with an average time to exhaustion of 24.73 ± 9.44 min [54] or a 20 min session of high-intensity interval training (HIIT) [59]. In the two remaining studies, moderate-intensity training sessions of 20 min [58] or 30 min [51] durations were applied. The effects of exercise intensity on the activation of GABA_A_-ergic receptors were reported in a recent study [91]. Observations from this study revealed significant post-exercise decrease of SICI after the high-intensity interval exercise but not after the low-intensity continuous exercise. A decrease in SICI after the high-intensity interval exercise was accompanied by increased facilitation of M1 corticospinal excitability (CSE) that remained unchanged following the low-intensity exercise intervention. Decreased SICI and increased CSE could suggest that downregulation of GABA_A_-ergic interneuronal circuits, observed after the high-intensity condition was mediated in part by a priming effect of exercise on glutamatergic network activity. However, to the best of our knowledge, evidence for a link among increased CSE, decreased SICI, and exercise-induced glutamatergic activity in M1 is still lacking.

#### 3.2.2. EEG

Evidence from the three ERPs studies [45,46,47] showed that acute exercise upregulated cognitive inhibition as seen by exercise-induced changes in the N140, P300, and N450 components. Findings from the study of Hsieh et al. [47] showed an overall increase in the magnitude of P300 and N450 components following exercise. Exercise-induced increase of the incongruent N450 amplitudes was negatively correlated with decreased reaction time in the incongruent conditions, suggesting that acute cardiovascular exercise may have facilitated cognitive inhibition processes that were related to conflict monitoring and attentional engagement. Similarly, findings from studies where the go/no-go task and the stop-signal task were used showed significant augmentation of the no-go N140 ERP component [45] and increased amplitude of the P300 component [46]. These effects were accompanied by a significant decrease of the amount of response errors in the go/no-go task [45] and improved response suppression (i.e., shorter SSRT) in the stop-signal task [46]. In all aforementioned studies, participants performed moderate acute exercise. Hence, moderate exercise intensity appears to facilitate both the cognitive and the motor aspects of response selection and response inhibition. Finally, findings from the study of Xie et al. [63] revealed no pre-to-post intervention in the magnitude of the P300 ERP, but the authors did observe a significant augmentation of late positive potential (LPP) components of the ERP at Fz. In line with this observation, Xie and colleagues concluded that high-intensity interval aerobic exercise was beneficial for improving attentional control and filter irrelevant information to achieve successful cognitive inhibition on the Flanker task.

One EEG study examined the effects of a single bout of physical exercise on cortical oscillations, arousal, and cognitive performance in young children [50]. The results show an overall increase in alpha oscillations (within the lower alpha frequency band) and significant decrease in beta oscillations of resting EEG after exercise. In this study, a greater pre-to-post decrease in the beta power band was found in the frontal cortex than in the central, parietal, and occipital electrode sites, suggesting a deactivation of frontal cortical areas. Similar to the findings of Mierau et al. [50] in children, modulations in brain oscillations during and/or after acute exercise in healthy adults were characterized by an overall increases of alpha and delta band oscillations and decrease of beta band oscillations [92,93,94]. These observations suggest, on the one hand, that acute exercise may generate an overall arousal activity that disappears immediately after the end of the exercise bout. On the other hand, findings from a recent study by Dal Maso et al. [95] showed that acute cardiovascular exercise tended to increase interhemispheric functional connectivity in both alpha and beta frequency bands among multiple pairs of electrodes. These pre-to-post effects of exercise on functional connectivity were more prominent for EEG activity in the beta frequency band, indirectly suggesting that acute exercise could increase GABA-ergic signaling. Indeed, findings from a recent pharmacological study on healthy humans [96] suggest that synchronization-desynchronization patterns in the alpha- and beta-bands can be modulated by administration of GABA-ergic agents. However, direct associations between these findings and specific inhibitory processes either at the cortical or system level require further investigation.

### 3.3. Association between Behavioral and Neurophysiological Measures

Observations consisting of behavioral data and neurophysiological (EEG, fMRI, and fNIRS) or biophysical (serotonin) measures were reported in seven studies [45,46,47,49,50,64,65]. In four of these studies [45,46,47,50], exercise-induced changes in EEG-outcome measures were reported (see Section 3.2.2). Findings from all EEG studies suggest that acute bout of cardiovascular exercise can facilitate activation of (pre)frontal and (pre)motor inhibitory brain networks and overall improve inhibitory control abilities. In addition, one study [47] showed that decreased interference RT on the Stroop task correlated with increased N450 amplitude in the incongruent trials following exercise. The authors suggested that this was an indication that cognitive inhibition following acute exercise may be accounted for, at least in part, by a more efficient conflict monitoring (as expressed by the increased N450 amplitude).

Two studies examined exercise-induced changes in brain activation during the performance of the go/no-go [49] or Stroop [64] task paradigms. Findings from the fMRI study of Mehren et al. [49] revealed no exercise-induced effect on brain activation or performance scores of the behavioral task (go/no-go) in a sample of healthy young adults. However, there were exercise-related increases in brain activation and behavioral task performance in age-matched individuals with attention deficit hyperactivity disorder (ADHD). Interestingly, ADHD patients with worse behavioral performance (i.e., lower correct inhibition rate) in the control condition showed stronger exercise-related increases in brain activation than ADHD patients with higher correct inhibition rate. These stronger exercise-related increases in brain activation were found in the insula and inferior frontal gyrus, middle and superior frontal gyri, pre- and postcentral gyri, supplementary motor area, supramarginal gyrus, mid cingulate cortex, inferior parietal and middle temporal regions, and the precuneus. These observations suggest that acute exercise may have stronger beneficial effects on brain activation in regions typically involved in tasks of response inhibition (e.g., inferior frontal cortex, insula, and supplementary motor area) when these functions are compromised.

In one study [64], exercise-induced changes in brain activation during the performance of a Stroop task were monitored with multichannel functional near-infrared spectroscopy (fNIRS). Decreased interference reaction time on the Stroop post-exercise was related to the oxy-Hb increase in dorsolateral prefrontal cortex (DLPFC). This finding is in line with observations from the ERP study of Hsieh et al. [47], supporting its involvement in regulation of cognitive inhibition. It is also evident the DLPFC is highly involved in resolving competition between possible movement alternatives and in organizing the mappings needed to perform a task. For example, two studies [14,15] showed that the magnitude of the release of interhemispheric DLPFC-M1 inhibition (i.e., disinhibition) during visually guided action preparation was associated with better performance of complex bimanual tasks. Further evidence for the involvement of DLPFC in the regulation of movement preparation and movement execution through inhibition/disinhibition of M1 came from recent studies where cortical excitability over the DLPFC was manipulated with repetitive TMS (e.g., [4,27]). Taken together, findings from all included studies using brain imaging methods suggest that acute exercise could improve top-down control of inhibition by increasing activation of prefrontal cortices. Finally, in one study [65], post-exercise changes in the performance of a Stroop task were examined against exercise induced changes in serum concentrations of serotonin (5-Hydroxytryptamine, 5-HT). Findings from this study show that acute intense aerobic exercise increased serum 5-HT concentrations and that this increase was associated with reduced Stroop interference effects.

## 4. Discussion

The observations from the reviewed studies suggest that a single bout of cardiovascular exercise improves inhibitory control. Behavioral effects include the decrease of interference effects [44,47,55,64,65] and improvement in response inhibition efficiency [45,46,48,49,52]. The immediate neurophysiological post exercise effects include more efficient task-related activation of the prefrontal and motor networks and greater recruitment of prefrontal brain resources (as indexed by analyses of ERP component amplitudes and fMRI/fNIRS data) [49,64].

Our focus is mainly on processes underlying response inhibition. Response inhibition is thought to be mediated by a network that includes the right inferior frontal gyrus (RIFG), the pre-supplementary motor area (preSMA), and the basal ganglia [16,17,18,35]. Therefore, our discussion focuses specifically on the effects of acute exercise on mechanisms involved in interactions between motor pathways and prefrontal-basal-ganglia pathways. From a perspective of cognitive inhibition, our focus is on processes related to top-down regulation of attention and conflict resolution that could involve recruitment of prefrontal and parietal pathways (e.g., [35]).

### 4.1. Perspectives on the Underlying Neural Mechanisms

The majority of the behavioral observations suggest that a single bout of exercise improves inhibitory control processes [44,46,47,48,50,52,55,57,64,65]. However, the underlying neural mechanisms are still under debate and are somewhat conflicting. On the one hand, findings from included EEG and neuroimaging studies show increased activation of prefrontal pathways that improves the efficiency of inhibitory control [45,46,47,49,50,64]. More specifically, these observations suggest that acute exercise could increase GABA-ergic signaling [50], facilitate cognitive inhibition processes that were related to conflict monitoring and attentional engagement [45,46,47], and intensify compensatory recruitment of prefrontal inhibitory pathways [49,64]. On the other hand, findings from the reviewed TMS studies [51,54,58,59] suggest an overall downregulation of GABA-ergic activity in M1 following the intervention that could potentially interfere with cortico-cortical inhibitory processes within this region. However, there is no clear line of evidence to suggest that post-exercise downregulation of GABA-ergic activity in M1 has a significant effect on inhibitory control through the activation of prefrontal pathways. Importantly, downregulation of GABA-ergic activity in M1 could have been induced by peripheral and/or central fatigue processes [54] and may therefore remain local.

An alternative explanation for increased efficiency of inhibitory control after the intervention despite the downregulation of GABA-ergic activity in M1 could be a shift in the balance between glutamate-induced excitation and GABA-mediated inhibition to a more excitatory state [97,98]. In line with this assumption, it is suggested that exercise-induced gains in inhibition may be mediated by increased glutamatergic activity. This assumption is consistent with existing literature on the role of cortico-striatal excitatory transmission in movement initiation and inhibition (e.g., [18]). Furthermore, findings from a recent study by Weerasekera et al. [34] support this assumption by pointing at a lateralized organization of the glutamatergic system in striatum. Specifically, Weerasekera et al. [34] showed that: (1) the glutamatergic activation in the left striatum predominantly accelerates the go process whereas glutamatergic activation in the right striatum proactively slows it down; and (2) the magnitude of this functional effect was significantly associated with higher levels of glutamate/glutamine (Glx) compounds in the striatum. Based on the aforementioned observations, we suggest that a single bout of cardiovascular exercise may act to improve both movement initiation and efficiency of prefrontal-basal ganglia inhibitory control by increasing the cortical concentrations of glutamate [99,100]. Other key processes and mechanisms fundamental for exercise-enhanced inhibitory control following cardiovascular training may include conversion of lactate to GABA through glutamine/glutamate/α-ketoglutarate pathways (e.g., [100,101]), thus increasing the availability of GABA and GABA tone in M1. Alternatively or additionally, exercise may also have a priming effect on CNS by transiently increasing blood flow to prefrontal or subcortical regions that regulate system inhibition such as prefrontal-basal ganglia pathways.

Finally, findings from recent studies by Hermans et al. [32] indirectly provide evidence for a role of GABA-ergic pathways in regulation of “stop” behavior, showing that longer SSRTs in older adults were associated with lower GABA concentration in the preSMA. Hermans et al. [32] proposed that GABA-ergic functioning in preSMA could partly contribute to the efficiency of reactive inhibition in older adults. Given the fact that exercise tends to increase GABA levels in the brain [100,101], we hypothesize that the increased availability of GABA in prefrontal network following exercise will improve inhibitory control. Future research may be conducted to examine the extent by which single bout of cardiovascular exercise could facilitated prefrontal–subcortical interactions for response inhibition in aging through exercise-induced increase in activation of GABA-ergic and glutamatergic neurotransmission.

### 4.2. Perspectives into Age-Related Effects

The results of the reviewed studies suggest that acute exercise can transiently improve the functioning of inhibitory pathways in young adults. An important question for aging research is how such exercise affects the aging brain. Indeed, age-related deficits in the ability to control cerebral inhibition may explain why healthy older adults have a range of motor and cognitive deficits, such as impaired coordination skills and declines in attention, concentration, and learning abilities (e.g., [19,102]). From a system level perspective, decreased cortical inhibition is expected to result in an activation spreading from task-relevant to task-irrelevant brain areas (e.g., [3,103]) which could lead to loss of network segregation and decrease in the specificity of functional interactions between brain regions engaged in a task [104,105,106]. For example, TMS studies showed that older individuals who present poor motor coordination skills also present a reduced modulatory capability of cortico-cortical inhibitory processes through the GABA_A_- and GABA_B_-ergic neurotransmission systems within the M1 ([5,41,107]; for a review, see [19]). Findings from other TMS studies using double-site TMS protocols (e.g., [14]) show that the decline in the ability to regulate inhibition in a task-related manner is attributed to decreased integrity of microstructural organization of WM tracts connecting the prefrontal areas with M1. In this respect, one may speculate that increased activity in prefrontal brain areas—as evident from fMRI [35,108,109,110,111]—is partly related to an increased recruitment of inhibitory motor pathways in high-performing older individuals in order to compensate for these structural declines. Finally, evidence from a recent study using resting state fMRI (rsfMRI) and ^1^H-MRS showed that lower GABA+ (i.e., GABA plus macromolecules/homocarnosine) levels in the sensorimotor cortex were associated with a weaker segregation of the sensorimotor network and that lower GABA+ levels and less segregated networks are associated with worse sensorimotor performance in older adults [104]. The finding that acute cardiovascular exercise increases brain activation of prefrontal inhibitory pathways during performance of an inhibition tasks in young healthy adults [64] or young individuals with ADHD [49] may provide indirect support for its potential beneficial effects on compensatory recruitment of inhibitory resources in the aging brain. Exercise-induced changes in neurotransmitter levels that appear to play an essential role in regulation of prefrontal neural activity (specifically but not exclusively GABA, glutamate, dopamine, and serotine) should be studies to examine their specific contribution for maintaining efficient inhibitory control over the lifespan. This presumption is supported, for example, by observations from recent research by McGregor and colleagues, showing that 12-week physical activity interventions were sufficient for improving interhemispheric inhibition [112] and for increasing segregation in the sensorimotor network [113]. Exercise-induced changes in the aforementioned neurophysiological measures were associated with improvements in motor dexterity and motor performance in general.

Based on the findings of the current review, it appears that: (1) a single bout of cardiovascular exercise can affect the balance between GABA-ergic and glutamatergic neurotransmission; and (2) a single bout of cardiovascular exercise improves inhibitory control at the system level. Therefore, it appears that this type of exercise has preferential impact on inhibitory processes in the aging brain. For example, one study [52] showed that moderate acute cardiovascular exercise facilitates response inhibition in a go/no-go task in middle-aged adults. However, the same intervention did not enhance a different task related to motor planning and eye-hand coordination and is not associated primarily with inhibitory control. The exact mechanisms behind this effect are not always clear, and, to the best of our knowledge, neurophysiological effects of a single bout of exercise on inhibitory control in older adults are not well understood.

### 4.3. Limitations

The ability to gain conclusive insights from the included literature is limited due to the heterogeneity in: (i) intensity, timing, and duration of interventions; (ii) type of inhibitory tasks; and (iii) neurophysiological outcome measured used. The standardization of exercise and testing protocols as well as consistency in the use of neurophysiological outcome measures will enable easier comparisons between studies [114,115]. In addition, the influence of confounding effects of fatigue, attention, emotional arousal, and motivation on both behavioral and neurophysiological aspects of inhibition are often not considered. Specifically, Chen et al. [116] suggested that researchers should characterize exercise training using variables such as frequency of training, intensity, duration, type of training, volume of training, and progression. For acute exercise, some of those variables are irrelevant (e.g., frequency and volume) but researchers would do well to include as many characteristics as possible of the exercise intervention. As Chen et al. [116] suggested, this would allow us to better understand which exercise characteristics affect brain function.

There are still considerable gaps in the literature examining the specific effects of acute exercise on the neurophysiology of inhibitory control in older adults. For example, there is an increasing use of TMS and ^1^H-MRS methods for studying the effect of aging on integrity of the GABA-ergic and glutamatergic neurotransmission systems on inhibitory control. However, only few studies have applied those methods to examine exercise-induced effects following a single bout intervention, possibly due to the transient nature of the post-exercise measures [58,99]. Finally, there may be a lack of appropriate lab settings for safe implementation of vigorous-to high-intensity acute exercise protocols in older adults.

## 5. Conclusions

The findings of the current review suggest that a single bout of cardiovascular exercise can enhance inhibitory control. Moreover, acute exercise appears to facilitate activation of prefrontal brain regions that regulate excitatory and inhibitory pathways (specifically but not exclusively the prefrontal-basal-ganglia pathways) which appear to be impaired in older age. Future research should investigate if the beneficial effects of acute cardiovascular exercise on GABA-ergic and glutamatergic neurotransmission processes observed in young adults could also be found in other populations.

## Figures and Tables

**Table 1:** A summary of the 22 studies that examined motor and cognitive inhibition after an acute bout of exercise.

Study	Participants	Task	Procedure and Exercise	Results	
				Performance	Neurophysiological Measures
**Include only behavioral measures**					
Abe et al., 2018 [44]	Healthy older adults (n = 26, mean age: 71.8 ± 4.7 years)	Japanese version of the color-word Stroop task	Before and after 10 min of chair exercise, including stepping, stretching, finger movements or rest (control) that were performed in a randomized order.	Pre-to-post reduction in Stroop interference effects in all three exercise conditions. Significant difference only between control condition and finger movement exercise.	
Joyce et al., 2009 [48]	Young adults (n = 10, mean age: 23 ± 2 years)	Stop-signal task (SST)	(1) during 30 min of cycling on an ergometer at 40% maximal aerobic power; (2) between 0 and 22 min from exercise termination; (3) between 30 and 52 min from exercise termination. Control: Seated on the ergometer without cycling (same participants tested on different days).	Go RT and SSRT decreased during exercise relative to rest. Beneficial effect of exercise on response execution (i.e., shorter Go RT) with maximum effect obtained around 40 min post exercise. No beneficial effect of exercise on SSRT.	
Netz et al., 2016 [52]	Middle-aged healthy active adults (n = 40, mean age: 51.88 ± 8.46 years).	Go/No-Go test and visuomotor skill test (Catch game test)	Before and after 25-min of treadmill walking at 60% HRR—experimental condition, or rest—control condition. Crossover design with 1 to 2 weeks between tests.	Pre-to-post improvement for both accuracy and RT immediately after the aerobic session was greater than the improvement in the control session. Performance scores returned to baseline levels at the follow-up test (30-min from the end of the aerobic session) No impact of exercise on visuomotor skills.	
Nouchi et al., 2020 [53]	Middle-aged (n = 29, mean age: 55.00 ± 3.59 years) and older (n = 30, mean age: 69.73 ± 5.32 years) healthy females.	Color-Word Stroop task for inhibition Digit symbol codding task for short-term working memory. Mood state measure (POMS2)	Two 12 min bouts of 12 resistance and aerobic exercises intervals followed by 6 min of stretching (intervention group) 30 min of rest (control group)	Improvements in inhibition performances measured with the Stroop task. Improvement in inhibition was associated with improvement of mood. No pre-to-post changes in working memory	
Pastor et al., 2019 [55]	Healthy adolescents (n = 35, mean age: 16.49 ± 0.79 years).	Color Stroop Test	Before and after a 20-min session of rest (theoretical physical education) or 20-min of physical activity at light/moderate or moderate/vigorous intensity (assessed with accelerometers).	Interference index of the Stroop task significantly increased from pre- to post-training session irrespective of intensity. No changes in the Interference index were observed after the rest session.	
Samani and Heath, 2018 [56]	Young adults (n = 26, age range: 19–26 years) divided into intervention group (n = 14) and passive controls (n = 12).	Anti-saccade task	Before and after a 10-min of cycling at moderate-to-vigorous intensity (60–85% HRmax; exercise group) or rest (passive control group).	Exercise group showed a significant decrease of the anti-saccade reaction time from pre- to post-exercise assessment. Exercise did not change the percentage of anti-saccade directional errors.	
Sibley et al., 2006 [57]	Young adults (n = 76, mean age: 22.50 ± 3.10 years)	Color Stroop Test and Stroop Negative Priming Test	Before and after a 20-min session of self-paced running/walking on a treadmill or rest in cross-over design.	Performance of the interference version of the Color Stroop task was faster following the exercise condition. Exercise intervention did not lead to a significant change in Stroop negative priming test performance.	
Sugimoto et al., 2020 [60]	Young men (n = 20, mean age: 21.0 ± 0.4 years)	Color-Word Stroop task	Before, immediately after, and follow-up tests (in 10-min intervals) for 30 min after repetitive exercise (R-EX) combining two bouts of 20 min of moderate-intensity exercise (60% VO_2 peak_) separated by 20 min sitting rest Single bout (S-EX) of 40 min moderate-intensity exercise (60% VO2 peak); cross over design	A significant decrease of the reverse-Stroop interference scores immediately after the end of exercise followed by a gradual return to baseline within 30 min post-intervention. No impact of exercise conditions on performance. No impact of exercise on mean arterial pressure, blood glucose levels, or blood lactate levels neither at the end of exercise nor at 30 min post-exercise recovery	
Wilke, 2020 [61]	Young adults (n = 35, mean age: 26.7 ± 3.6 years) divided into three groups	Color-Word Stroop Test Trail Making Test (TMT) Digit Span Test (DST)	Before and after 15 min of HIFT Including 30 sessions of 20 s all-out training bouts and 10 s rest (HIFT group) 15 min of treadmill walking at 60% HRR (WALK group) 15 min of rest (Control group)	HIFT improved inhibitory control (Stroop interference effect decreased) and increased short-term/working memory (on the DST). No pre-to-post changes for WALK and Control	
Wilke and Royé, 2020 [62]	Young adults (n = 24, mean age: 26 ± 4 years)	Color-Word Stroop Test Trail Making Test (TMT) Digit Span Test (DST)	Before and after 15 min of FCT including 30 sessions of 20 s training bouts and 10 s rest periods at high (maximal effort), moderate (40–59% HRR) or low (20–39% HRR) intensity (cross-over design).	Exercise intensity did not significantly affect the magnitude of pre-to-post changes in performance. Trend towards an improvement on Stroop and TMT after moderate and high intensity FTC	
**Include only neurophysiological measures**					
Mooney et al., 2016 [51]	Young adults (n = 10, mean age 23 ± 2 years)		TMS-based assessments of corticomotor excitability (CME), cortical silent period (cSP), intracortical inhibition (SICI, LICI), and late cortical disinhibition (LCD). Measurements were taken before and 10–50 min after a 30-min session cycling at workload corresponding to 60% of peak VO_2_) or rest (seated stationary on the cycle ergometer).		TMS: A transient decrease of LICI (downregulation of GABA_B_ receptor) up to 20 min post-exercise. No significant changes in all other TMS measures.
Singh et al., 2014 [58]	Young adults (n = 12, average age: 28 years)		TMS-based assessments of CME, SICI, LICI, and intracortical facilitation (ICF) before and after a 20-min of cycling at 65–70% of age-predicted HRmax.		Pre-to-post decrease of SICI (downregulation of GABA_A_ receptors) and LICI (GABA_B_ receptors). Significant changes relative to pre were found only 30 min following exercise and only for SICI). Significant pre-to-post increase of ICF (significant change relative to pre-level was observed only imminently after the end of the exercise). No exercise-induced changes in CME.
Stavrinos and Coxon, 2017 [59]	Healthy adults (n = 24, average age = 23.63 ± 4.38 years; age range: 19–41 years).	Learning of a visuomotor skill	TMS-based assessments of corticomotor excitability, SICI and LICI before and after a 20-min session of high-intensity cycling (exercise group) or rest (control group).		Pre-to-post decrease of SICI (downregulation of GABA_A_ receptors). Exercise-induced enhancement of motor skill consolidation. Significant positive association between downregulation of GABA_A_-receptor (reduced SICI) and offline skill improvement during retention. No exercise induced effect on corticomotor excitability or GABA_B_ inhibition (LICI).
**Include both behavioral and neurophysiological measures**					
Akatsuka et al., 2015 [45]	Young healthy adults (n = 10, mean age: 19.8 years)	Go/no-go task	Before and after a 15-min treadmill running at 50% peak oxygen intake + 5 min rest Control: same participants, 20-min period of rest.	RT and % missed response decreased in post- as compared to pre-tests in both groups, but no significant group or time effects were seen.	EEG recordings from seven scalp positions (Fz, Cz, Pz, FC3, FC4, PC3, and PC4). Significant augmentation of the no-go-N140 ERP components at Fz and Cz after performance of the moderate aerobic exercise.
Chu et al., 2015 [46]	Young healthy adults (n = 21, age range: 19–24 years)	Stop-signal task (SST)	Before and after a 30-min exercise session on a treadmill (5 min warm-up, 20 min exercise at 65–75% HRmax, and 5 min cool-down). Control: same participants, reading exercise-related articles for 30-min (tests conducted at least 24 h apart).	Stop-signal response time (SSRT): Exercise faster than control No changes in the go response time (go RT) neither after the exercise nor the control sessions.	EEG recordings from 32 scalp position. ERPs were time-locked to the onset of the stop signal. Significant augmentation of the P3 component at Pz, Cz and Fz and a longer parietal P3 latency after the exercise session.
Hsieh et al., 2018 [47]	Young adult males (n = 24, mean age: 24.0 ± 3.1 years). Older adult males (n = 20, mean age: 70.0 ± 3.3 years).	Modified Stroop Color-Word test	Before and 15 min after a 20-min bout of moderate intensity aerobic exercise (60–70% of HR reserve) on a treadmill. Exercise was preceded/followed by 5 min warm-up/cool-down. Control: same participants, watching a video relating to sport science for 30-min while sitting quietly (tests conducted at least 96 h apart).	RT were faster in exercise group than control during the congruent and incongruent trials. Stroop interference effects following exercise were smaller relative to those observed in the control group irrespective of age.	EEG recordings from 64 scalp position (10–20 system). P3 and N450 components showed larger amplitudes in exercise relative to controls irrespective of age. Exercise-induced changes in response time interference were negatively correlated with exercise-induced changes in incongruent N450 amplitudes.
Mehren et al., 2019 [49]	Young adults (n = 23, mean age: 29.5 ± 7.0). (note: the study included young adults with ADHD as well—however, observations from these participants were not included in this review)	Go/No-go task	Before and immediately after a 30-min moderate (50–70% HR max) exercise session on a cycle ergometer. control session: 30 min of watching a movie.	No effect of exercise on performance the Go/No-go task.	fMRI: Significant effect of exercise on brain activation during the Go/No-go task during correct inhibition. BOLD signal responses within the left superior occipital gyrus (SOG), right precuneus (PCUN), and left supramarginal gyrus decreased in the exercise compared to the control condition. No significant associations with performance measures.
Mierau et al., 2014 [50]	Young children (n = 10 males, mean age: 5.8 ± 0.4 years)	Children’s version of the determination test (DTC).	Before and after a 45 min exercise (30 min of movement games followed by 15 min of a soccer match) or control condition (60 min of seated rest) in cross-over design.	Significant decrease in RT and significant increase of correct responses from pre to post but no significant effects for condition.	EEG: Significant increase of alpha-1 and significant decrease of beta-1 and beta-2 power bands after exercise. Pre to post changes in regional beta-1 and beta-2 power were detected in prefrontal but not in parietal, central, or occipital regions.
O’Leary et al., 2018 [54]	Young adults (n = 18, mean age: 26 ± 5 years).	MVC task	TMS-based assessment of cortical silent period (cSP) and long intracortical inhibition (LICI) before and after high-intensity exhaustive cycling session. Assessment of peripheral and central fatigue with motor nerve stimulation (MNS) and TMS.	Post exercise reduction in knee extensor MVC torque. Post exercise increase in levels of peripheral and central fatigue.	Significant decrease of LICI (16 ± 14%) and cSP duration (−31 ± 28 s) due to exercise (both indicating downregulation of GABA_B_ receptors). No post-exercise changes in MEP.
Xie et al., 2020 [63]	Obese young adults (n = 16, mean age: 24.50 ± 5.09 years)	Flanker task	Before and after 20 min of high intensity interval exercise (HIIE) including 10 sessions of ± 1 min of 80–90% maximal HR (HRmax) interspersed by 1 min of 50–65% HRmax active relax. (5 min warmup/cool down) Control condition: 30 min of rest (cross-over design).	Shortening of RT after HIIE in both congruent and incongruent conditions of the Flanker task. No exercise induced changes in response accuracy.	EEG recordings from 64 scalp position. ERPs were monitored 200 ms before to 1500 ms after the stimulus onset. No significant changes in the P3 amplitude. Significant augmentation of late positive potential (LPP) components of the ERPs at Fz after HIIE
Yanagisawa et al., 2010 [64]	Young adults (n = 20, mean age: 21.5 ± 4.8 years)	Stroop-interference task	Before and 15 min after a 10-min moderate aerobic exercise (50% Vo2 peak) or 25 min of rest (a cross over design).	Shortening of Stroop-interference reaction time after the acute exercise.	Findings from multichannel near-infrared spectroscopy (fNIRS) revealed increased activation of the left DLPFC during the Stroop interferences task after the acute exercise. Stroop interference-related increase in the activation of DLPFC and shortening of Stroop-interference reaction time after the acute exercise were coincided.
Zimmer et al., 2016 [65]	Young adults (n = 121, mean age: 23.8 ± 3.6 years).	Stroop task	Before and after a 30-min session of aerobic exercise at low intensity (LI, n = 30; 45–50% of HRmax) moderate intensity (MI, n = 30; 65–70% of HRmax), or high intensity, (HI, n = 30; 85–90% of HRmax), or 30 min of rest (CG, n = 31).	Significant difference for Stroop parameter reading and a tendency for reverse Stroop effect. Significant pre-to-post decrease in reaction time for color reading task between the LI and HI.	Higher Serotonin (5-HT) serum concentration in the HI group after exercise. Higher 5-HT serum levels were associated with overall decreased reaction time on the Stroop task after exercise.

5-HT, 5-hydroxytryptamine; BOLD, Blood Oxygenation Level Dependent; CME, corticomotor excitability; cSP, cortical silent period; DLPFC, Dorsolateral Prefrontal Cortex; DTC, Determination Test; EEG, Electroencephalogram; ERP, Event Related Potential; FCT, Functional Circuit Training; fMRI, Functional Magnetic Resonance Imaging; fNIRS, Functional Near-infrared Spectroscopy; GABA, Gamma Aminobutyric Acid; HIFT, High Intensity Functional Training; HR, Heart Rate; ICF, Intracortical Facilitation; LCD, late cortical disinhibition; LICI, Long-interval Intracortical Inhibition; MNS, Motor Nerve Stimulation; MVC, Maximal Voluntary Contraction; PCUN, precuneus; RT, Reaction Time; SICI, Short-interval Intracortical Inhibition; SOG, superior occipital gyrus; SSRT, Stop signal response time; SST, Stop Signal Task; TMS, Transcranial Magnetic Stimulation.

## Data Availability

Not applicable.
